# Psychometric properties of the Persian language version of the Female Sexual Function index among postmenopausal women

**DOI:** 10.4274/jtgga.2017.0131

**Published:** 2018-11-15

**Authors:** Masaudeh Babakhanian, Masumeh Ghazanfarpour, Mona Najaf Najafi, Fatemeh Rajab Dizavandi, Talat Khadivzadeh, Minoo Safaei, Mehdi Jabbari Nooghabi

**Affiliations:** 1 Social Determinants of Health Research Center, Semnan University of Medical Sciences, Semnan, Iran; 2 Department of Midwifery, Kerman University of Medical Sciences School of Nursing and Midwifery, Kerman, Iran; 3 Department of Community Medicine, Imam Reza Clinical Research Units, Faculty of Medicine Mashhad University of Medical Sciences, Mashhad, Iran; 4 Department of Community Health and Psychiatric Nursing, School of Nursing and Midwifery, Mashhad University of Medical Sciences, Mashhad, Iran; 5 Department Professor of Statistics, Ferdowsi University of Mashhad, Mashhad, Iran

**Keywords:** Female Sexual Function index, psychometric properties, confirmatory factor analysis

## Abstract

**Objective:**

The present research aimed to evaluate the psychometric properties of the Persian language version of the Female Sexual Function Index (FSFI) among postmenopausal women.

**Material and Methods:**

This secondary analysis examined 402 healthy postmenopausal Iranian women presenting to healthcare centers across Iran. The sampling method was convenience sampling. The translation of the FSFI and its cross-cultural adaptation were conducted under the guidelines proposed by Beaton. The reliability (Cronbach’s alpha coefficient and test-rest reliability) and construct validity confirmatory factor analysis) were assessed. Model fitting index [such as the root mean square error of approximation (RMSEA), the Goodness of Fit Index (GFI) and the Comparative Fit Index (CFI)] was calculated.

**Results:**

The mean age of the study participants was 53.63±7.8 years. Test-retest reliability was high for both the entire scale (r=0.964; p<0.001) and its six dimensions (0.76-0.94; p<0.001). The Cronbach’s alpha of the entire scale and its dimensions was greater than 0.80. The original six-factor was used, which showed a relatively poor fit (χ^2^=667.054; p<0.001; χ^2^/df=4.86; GFI=0.92; RMSEA=0.098; GFI=0.85). After adding three correlated error terms to the six-factor model, an acceptable fit was obtained (χ^2^=470.542; p<0.001; χ^2^/df=3.51; CFI=0.95; RMSEA=0.079; GFI=0.89).

**Conclusion:**

According to our results, the FSFI tool indicated a satisfactory fit for a six-factor model, as similar to the original English version, for use in clinical practice and research regarding healthy postmenopausal Iranian women. More research needs to be conducted on this scale to assess all of its psychometric properties.

## Introduction

Human sexuality is an integral role in everyone’s life (1,2). A growing body of literature has examined sexual issues in older adults. The World Association for Sexual Health changed its slogan to “Sexual Health for All” (2). Menopause negatively affects nearly all the dimensions of sexual function, including lubrication, pain, and orgasm (3). Almost 50% of postmenopausal women in the United States of America are estimated to have sexual dysfunction (4). Sexual problems negatively affect quality of life and personal relationships (5) and can even lead to divorce (6). 

Based on recent rapid advances, valid instruments are required for diagnosing and treating female sexual dysfunction (FSD) (7). Among these, Rosen et al. (8) developed the Female Sexual Function index (FSFI) as a multidimensional self-report tool to detect the dimensions of female sexual function. The original English version of this instrument exhibited an excellent internal consistency (a=0.82 or higher) and a proper test-retest reliability during a two-week interval (r=0.79-0.88). The tool is well capable of discriminating between healthy women and women with sexual dysfunction, suggesting its good discriminant validity. The psychometric properties of this instrument have been assessed in different languages and cultures (9,10,11,12).

Almost all tools have been designed and developed to measure the different dimensions of sexual function in younger people. Research on sex and sexuality in older women may encounter difficulties due to the sensitive nature of the subject (13), especially in Iran, because talking about sexual relations is a taboo, in particular among older women, so there has been limited research on sexuality in the elderly population (13). Changes are induced with aging and menopause such as changes in sexual response, the female genitalia, orgasmic function, and sexual hormones (11). 

A review of the literature in national and international databases yielded only two studies assessing the psychometric of the FSFI in Iran (14,15). The psychometric properties of this instrument have never been assessed in postmenopausal women who are mostly at risk of FSD as compared with their younger counterparts (16). The objective of the current study was to evaluate the psychometric properties of the FSFI in Iranian postmenopausal women.

## Material and Methods

This secondary analysis combined the data collected in two previous cross-sectional studies conducted at different times in Iran. One study was conducted in the Semnan province in northern-central Iran (sample 2=202) and the other one in the city of Torbat-e Heydarieh, in Mashhad province (sample 1=200). The ethics committee of Torbat-e Heydarieh University of Medical Science and Semnan University of Medical Science approved the two previous studies. The patients completed informed consent forms voluntarily. Menopause was defined as being older than 45 and having had amenorrhea for at least one year. The subjects with severe medical diseases or psychiatric disorders were excluded from the study.

### Translation and cross-cultural adaptation

The translation of the FSFI and its cross-cultural adaptation were conducted under the guidelines proposed by Beaton et al. (17). A team of two bilingual translators whose mother tongue was Persian and who were fluent in English translated the English scale into Persian. Two native English speakers back-translated the Persian version into the English scale. The translators and the researcher synthesized the two translations into a single version and wrote a report about the synthesis process. An expert committee of translators, two health professionals, and one expert in psychometrics consolidated all the translations into the pre-final version, which was tested on 40 women.

### Assessment of content validity

The quantitative content validity of the Persian scale was assessed via the Content Validity index (CVI) and the Content Validity Ratio (CVR). An expert panel of eight sexual and reproductive health specialists and gynecologists assessed the content validity of the scale, which was reported to be excellent based on the CVR and the CVI.

### The Female Sexual Function index

This is a brief, multidimensional, self-reporting index used to assess sexual dysfunction, consisting of 19 items within six dimensions rated on a Likert scale from 0 to 5 or from 1 to 5, including desire (items 1-2), subjective arousal (items 3-6), lubrication (items 7-10), orgasm (items 11-13), satisfaction (items 14-16), and pain (items 17-19). Zero scores belong to those reporting no sexual intercourse within the past four weeks. Higher scores (in total, for the items or for the dimensions) indicate less sexual dysfunction (8).

### Reliability and validity assessment

Cronbach’s alpha coefficient was used to calculate the internal consistency of the entire FSFI and relevant dimensions, including fair consistency if reported as 0.7, moderate if reported as 0.7 to 0.8, and excellent if reported as 0.9 and over (18). Pearson’s r coefficient was applied to evaluate the test-retest reliability, which was fulfilled within a two-week interval on a sub-sample of 40 women. 

The FSFI factor structure was also evaluated using the confirmatory factor analysis (CFA), conducted on a sample of 402 postmenopausal women. The inter-correlation between the dimensions of the scale and the correlation between the entire scale and its dimensions were estimated using Pearson’s correlation coefficient.

### Statistical analysis

The CFA was performed in AMOS-18 (http://www3.ibm.com/software/products/en/spss-amos) using the maximum-likelihood method for parameter estimation. The root mean square error of approximation (RMSEA), the goodness of fit index (GFI) and the comparative fit index (CFI) determined the modified eight-factor data model. Values above 0.9 were recommended for CFI and GFI, and below 0.08 for RMSEA (19,20). The ratio of chi-square to the degree of freedom (χ^2^/df<5) was found to be acceptable by Marsh and Hocevar (21).

## Results

Table 1 presents the demographic characteristics of the study participants, including 402 postmenopausal women with a mean age of 53.63±7.8 years. 

More than half of the subjects were illiterate or had primary school education and only 6% had university education. A total of 78.6% had more than two children. There were no missing data because questions were immediately checked after they were returned by the participants.

### Confirmatory factor analysis

**Figure 1:**
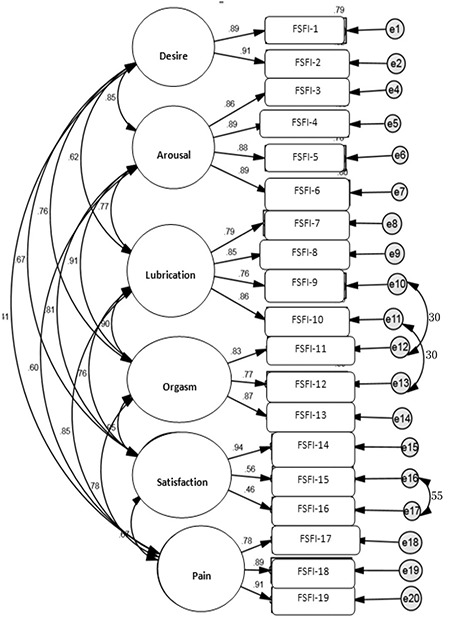
Six covariated factor model *p<0.05; FSFI: Female Sexual Function index*

The original English six-factor [Rosen et al. (8)] was used, which showed a relatively poor fit (χ^2^=667.054; p<0.001; χ^2^/df=4.86; GFI=0.92; RMSEA=0.098; GFI=0.85). After adding three correlated error terms to the six-factor model based on the largest modification indices provided by AMOS, an acceptable fit was obtained (χ^2^=470.542; p<0.001; χ^2^/df=3.51; CFI=0.95; RMSEA=0.079; GFI=0.89) (Table 2). The correlated errors were between item 10 and 12, between 15 and 16, and between 9 and 11. The factor loading of these items was in a range of 0.46 to 0.94 (Figure 1). The chi-square value, however, remained significant, which could be attributed to the large sample size (Table 2). The strongest correlated error was observed between item 15 and item 16. 

Although an acceptable fit with the data was found for the six-factor model, we also tested other models suggested in other studies (10,14,22). Initially, the first-order, one-factor model was tested to evaluate if of 19-item FSFI could be included into a single factor. This model revealed poor fit with the data (χ^2^=1832.362; p<0.001; χ^2^/df=12.05; GFI=0.57; RMSEA=0.166; CFI=0.75). The five-factor model was tested and showed a poor fit to the data (p<0.001; χ^2^/df=5.67; CFI=0.9; GFI=0.823; RMSEA=0.1) (Table 2).

### Reliability

The analysis was performed using the data obtained from sample 1. A total of 40 subjects were asked to visit again to complete the FSFI. The degree of agreement between the two assessments was measured within a two-week interval and was found to be high for both the entire scale (r=0.964; p<0.001) and its six dimensions (0.76-0.94; p<0.001) (Table 3). The internal consistency of the scale was determined using the Cronbach’s alpha coefficient. As can be seen in Table 3, the Cronbach’s alpha values of the FSFI and its dimensions, which were greater than 0.80 for almost all the dimensions and for the entire scale, revealed excellent internal consistency reliability (Table 3).

### Inter-correlations

There were slightly high significant correlations between the different dimensions of the FSFI, among which the strongest correlation between orgasm and satisfaction (0.938), and the weakest between pain and desire (p=0.409). The p values became significant for all the dimensions. The correlations were significant and positive and greater than 0.4 between all the items (Table 4).

## Discussion

The objective of the current study was to evaluate the psychometric properties of the FSFI in Iranian postmenopausal women. The main conclusion of this study was that six-factor models with three correlated error terms were a good fit to the data, but other models (five-factor model, second-order, six-factor model, and one-factor model) showed a poor fit to the data.

Menopause is an important stage during the lifetime of all women (11). All domains of sexual function (orgasm, lubrication, desire and sexual pain) are likely to be negatively affected by menopause (23). Sexual problems have been frequently reported in Asian countries, such as Korea and Iran, due to the conservative nature of sex and sexuality in such countries (11,23). Despite the importance of sexuality for adults in Korea, only 2% of women and men consult health providers (11). According to a qualitative study, many Iranian postmenopausal women do not discuss their sexual problems with healthcare providers for a variety of reasons, including traditional, cultural, and religious beliefs (13).

The FSFI instrument has been developed for both clinical practice and research. It has been translated into various languages and also validated in different samples of women. Nevertheless, it has never been validated for use in postmenopausal women despite its extensive use in research. In terms of reliability, the FSFI shows excellent internal consistency. As for construct validity (CFA), six-factor models indicated a good fit with the data. This result is consistent with the findings of Rosen et al. (8) and Opperman et al. (24), who also found that a six-factor model of the FSFI with 19 items appropriately fitted with data.

Of significant correlation found between the dimensions of the FSFI, the strongest correlation was reported between orgasm and satisfaction (r=0.938). Takahashi et al. (10) in Japan and Nowosielski et al. (25) in Poland observed the strongest correlation between lubrication and arousal. In a study by Vallejo-Medina et al. (26) in Spain, the strongest correlation existed between satisfaction and arousal. Fakhri et al. (14) in Iran also found the strongest correlation between lubrication and desire. The difference between the present study and the other studies cited may be due to the study population, i.e. postmenopausal women, who often have some degree of vaginal atrophy and vaginal dryness (27). 

The present study found excellent internal consistency for the entire scale and a good internal consistency for all its dimensions. These findings are in line with previous studies, in which the internal consistency of the scale was high to excellent with Cronbach’s alpha ranging from 0.84 to 0.95 in the Japanese (10), 0.83-0.94 in Chinese (9), 0.85 to 0.94 in Arabic (28), and 0.88 to 0.96 in Iranian versions regarding women of reproductive ages (14). 

The two-week test-retest reliability was found to be high in the entire scale and associated dimensions, which is consistent with the findings of other studies. For example, Fakhri et al. (14) in Iran, Takahashi et al. (10) in Japan, and the assessment of the original English version (8) showed good or excellent test-retest reliabilities for the scale. In the study by Nowosielski et al. (25), good internal consistency and test-retest reliabilities of the scale were exhibited among both healthy subjects and women with sexual problems (29). 

According to the present model, the best fit was observed in the six-factor model, although some studies have found the five-factor model to have better psychometric properties (26).

### Study limitations

This study had several limitations. First, it assessed only healthy menopausal women with no serious diseases who were selected through convenience sampling; the generalization of the results for all Iranian women in different subgroups (including reproductive ages or sexual problems or diseases) should therefore be pursued with caution. Second, we assessed neither the discriminant validity of the study scale nor the measurement invariance of menopause status. Similar studies are required to assess the other psychometric properties of this scale. 

According to our results, the FSFI tool indicated a satisfactory fit for six factor model, similar to the original English version, for use in clinical practice and research regarding healthy postmenopausal Iranian women. More research needs to be conducted on this scale to assess all of its psychometric properties.

## Figures and Tables

**Table 1:**
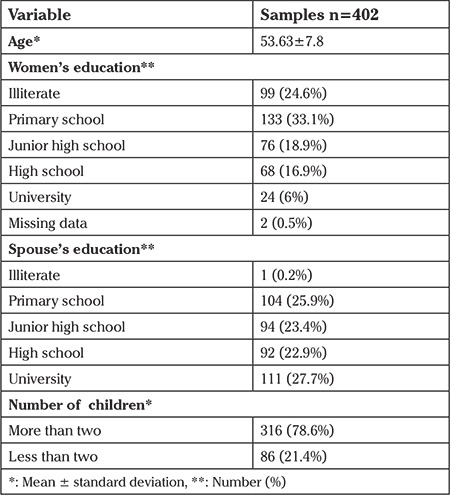
Participants’ characteristics

**Table 2:**
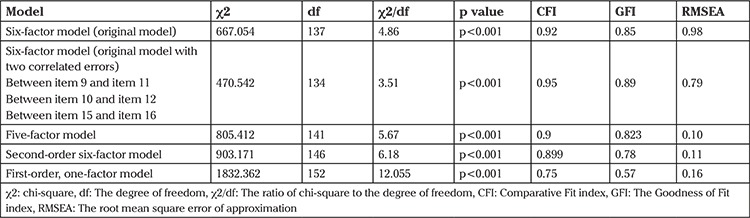
The fit indices of the original model and the five other models

**Table 3:**
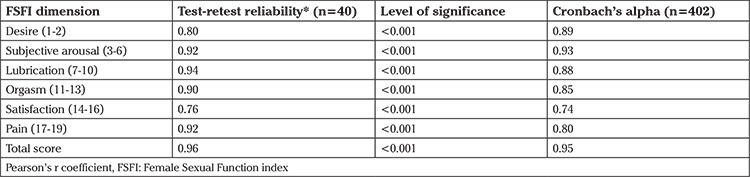
The test-retest and Cronbach’s alpha of the Persian version of the FSFI

**Table 4:**
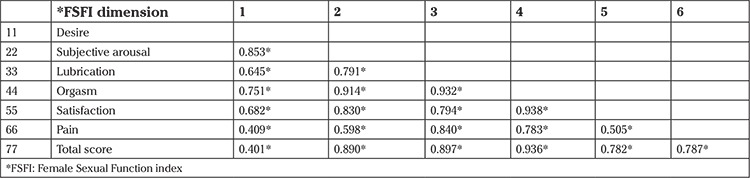
The correlation between the FSFI dimensions
